# Social Media Posts by Recreational Marijuana Companies and Administrative Code Regulations in Washington State

**DOI:** 10.1001/jamanetworkopen.2018.2242

**Published:** 2018-11-16

**Authors:** Megan A. Moreno, Aubrey D. Gower, Marina C. Jenkins, Josh Scheck, Jaymin Sohal, Bradley Kerr, Henry N. Young, Elizabeth Cox

**Affiliations:** 1 Department of Pediatrics, University of Wisconsin-Madison, Madison; 2 Undergraduate Student, Department of Pediatrics, University of Washington, Seattle; 3 School of Pharmacy, University of Georgia, Athens

## Abstract

**Importance:**

Recreational marijuana use was legalized in the state of Washington in 2012, and the Washington Administrative Code (WAC) 314-55-155 was implemented to limit the exposure of youths to marijuana advertisements.

**Objectives:**

To evaluate the content of social media posts by marijuana companies and to assess the compliance of these posts with WAC regulations.

**Design, Setting, and Participants:**

In a cross-sectional study, a content analysis of 1027 social media posts was conducted to identify and assess compliance of WAC regulations with the business pages of recreational marijuana companies located on Facebook and Twitter platforms in the state of Washington from December 1, 2015, through November 30, 2016.

**Main Outcomes and Measures:**

Application of coding based on WAC regulations to focus on the prohibition of language that promoted the overconsumption of marijuana, that described its curative or therapeutic benefits, or that was designed to appeal to youths; and the requirements for warnings about intoxication, driving, health risks, and age restrictions for marijuana.

**Results:**

Of the 1027 posts on Facebook and Twitter from business pages of 6 marijuana companies, Facebook followers ranged from 342 to 1592 persons and Twitter followers ranged from 374 to 2915 persons per company. Findings for WAC regulations included 17 posts (1.7%) that encouraged overconsumption; 137 posts (13.3%) that promoted therapeutic benefits; and 9 posts (0.01%) that appealed to youths. Requirements for warnings addressing intoxication, driving, health risks, and age restrictions were present on 110 posts (10.7%). Some businesses repeatedly violated particular regulations; 7 of 17 (41.2%) posts encouraging overconsumption derived from 1 marijuana company.

**Conclusions and Relevance:**

Most social media posts by marijuana companies were consistent with WAC regulations that prohibit particular messages, but few companies provided required warning messages. Findings can be used toward implementation strategies for marijuana prevention to address these influential media messages.

## Key Points

**Question:**

How often are recreational marijuana companies adhering to the state of Washington Administrative Code regulations when posting product promotion messages on social media?

**Findings:**

This cross-sectional content analysis of 1027 posts on Facebook and Twitter platforms evaluated the social media content of business pages from 6 recreational marijuana companies. Violations of regulations regarding prohibited content were present for between 2% and 13% posts across regulation categories; required warnings were present on only 11% of posts.

**Meaning:**

Social media are influential and accessible platforms for youths in which recreational marijuana companies promote prohibited content and avoid required health warning messages.

## Teaser

This cross-sectional content analysis assesses adherence of social media content of recreational marijuana companies in the state of Washington to Washington Administrative Code regulations.

## Introduction

The state of Washington passed Initiative Measure No. 502 in 2012, which legalized recreational marijuana use for persons older than 21 years.^1,2^ Legalization of marijuana has raised concerns about access to marijuana by youths younger than 21 years, and increases in the underage consumption of marijuana.^2^ Adolescents are a crucial at-risk population for marijuana use; rates of use among adolescents are approximately 15% for use within the last 30 days and 30% for lifetime use.^3^ Consequences of marijuana use include academic difficulties such as school dropout, psychiatric impairment including memory loss, and progression of use to other drugs.^4,5^

The legalization of recreational marijuana may increase the exposure of youths to and influence from messages promoting marijuana use. While direct advertising of marijuana on social media remains illegal, marijuana companies can create profiles called “business pages” on Facebook and Twitter. Business pages provide a continuous presence on social media at no cost. The messages about marijuana generated on these pages may be influential to youths. Previous research results showed that exposure to content generated by a tobacco company can influence individuals about the appeal of smoking.^6,7^

Marijuana companies can use social media business pages to reach potential consumers who can “Follow” the business page. Followers can engage with the content on the business page by endorsing it via Likes or Favorites, or by sharing it on their own web page via Shares or Retweets, thus increasing the reach of the content. Marijuana companies on social media can engage with individuals to achieve ongoing exposure of youths to messages about marijuana. Furthermore, marijuana messages on social media may exert influence through interactive strategies. Examples are described in a *Forbes* article by Weed^8^ including strategies that involve building online communities through hashtags and that use visual media toward “redefining the stoner stereotype.”

There are few restrictions on the social media content generated by marijuana companies in the state of Washington. The Washington State Liquor Control Board Washington Administrative Code (WAC) 314-55-155 is the main creator and enforcer of advertising restrictions for marijuana companies. The 2015 WAC regulations stated that marijuana companies cannot advertise using language that promotes overconsumption of marijuana; that describes its curative or therapeutic benefits; or that is designed to appeal to youths.^9,10,11^ Messages must include warnings about the risks of intoxication; about driving under the influence of marijuana; about health risks from using marijuana; and about prohibiting underage use. Explicit social media information was as follows: “Please use social media with caution and be mindful not to appeal to, or solicit, viewers under the age of 21. If possible, please restrict views to adults age 21 and older.”^12^ Social media represents a platform for messages, interaction, and promotions; direct advertisements for marijuana remain illegal under federal guidelines. It is unclear whether businesses follow these WAC regulations in their presence on social media. If not, social media may be a way to bypass advertising restrictions on social media platforms that are extremely popular among youths.^13^

The development, application, and enforcement of policies designed to prevent the use of marijuana in youths are essential to consider at the early stages of legalization of marijuana for recreational use. An understanding of the current state of advertising policies applied to social media is important and timely. The purpose of this study was to evaluate the adherence to WAC regulations among the social media business pages of recreational marijuana companies in the state of Washington. In addition, to understand whether the presence of the required WAC warnings reduced the engagement of users with these marijuana business posts, we tested whether social media posts with WAC warnings had decreased the endorsement of users (via Likes/Favorites and Shares/Retweets) compared with social media posts without WAC warnings.

## Methods

This study used content analysis to evaluate social media posts on the public Facebook and Twitter business pages of recreational marijuana companies in the state of Washington. Similar to previous studies, this social media content analysis involves a structured, detailed evaluation of content that was posted on social media sites.^14,15,16,17,18^ The evaluation of social media covered 1 year—from December 1, 2015, through November 30, 2016. This study was approved by the institutional review board of Seattle Children’s Hospital, Seattle, Washington, and followed the Strengthening the Reporting of Observational Studies in Epidemiology (STROBE) reporting guidelines for cross-sectional studies. The institutional review board of Seattle Children’s Hospital determined this study to be exempt from participant informed consent because the review of publicly available information focused on companies and was not human patient research. This article contains no studies with human or animal participants that were performed by any of the study authors.

### Profile Identification

Data for this study were obtained from 2 social media platforms, Facebook and Twitter, which are among the most popular social media sites for both youths and businesses using social media.^13^ We initially identified potential marijuana businesses using several approaches, including searching on Facebook, Twitter, and the marijuana business locator website Weedmaps.^19^ We then determined whether the business was present on both Facebook and Twitter. If so, we evaluated the following inclusion criteria: (1) the business focused on retail recreational marijuana products; (2) the business was located in the state of Washington with the goal of 1 business per city or town; and (3) the business has maintained both social media profiles since 2015, thus a full year of content was available.

We identified 38 recreational marijuana businesses in Washington with a social media presence on Facebook or Twitter. Our flowchart and exclusions are described in the Figure. Each business was assigned a letter (eg, company A) for our data collection and reporting.

**Figure.**
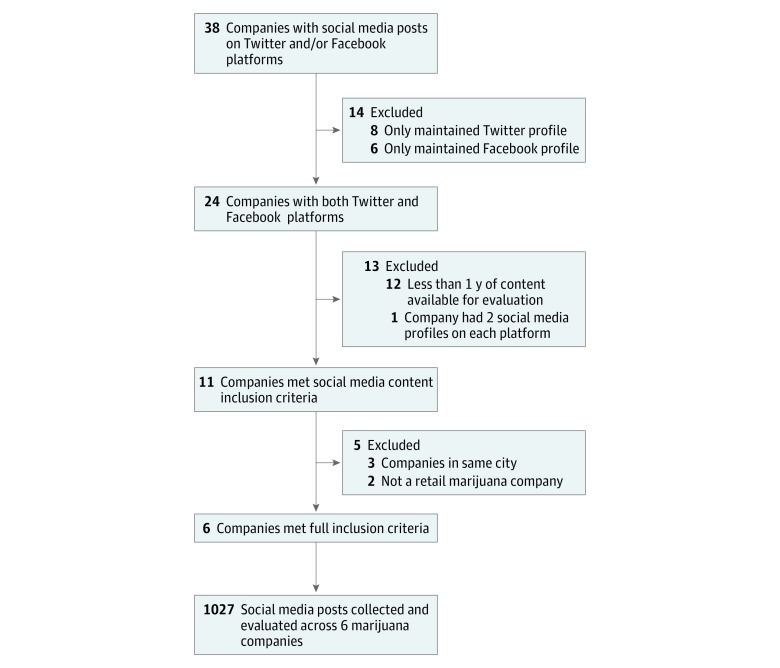
Flowchart of Recreational Marijuana Companies With Social Media Pages in the State of Washington

#### Codebook Development

The codebook was a priori guided by the WAC 314-55-155 regulations. These WAC regulations informed coding to identify marijuana business posts that represented endorsement of (1) overconsumption, (2) curative or therapeutic benefits, and (3) appeal to youths; as well as the identification of warnings (4) of the product’s intoxicating effects, (5) of avoiding driving while using marijuana, (6) of health risks, and (7) of product use limited for people older than 21 years.

#### Pilot Coding and Codebook Validation

For marijuana businesses not included in this research, we conducted pilot coding to test and refine the codebook to represent the key elements of the WAC regulations. A total of 6 rounds of pilot coding, followed by codebook revision or clarification, were conducted. Our pilot evaluations revealed that companies had varied the frequencies of social media posting. Companies often repeated previous posts during workday hours and were more likely to generate new content for afternoon or evening posts.

To evaluate our coding process and ensure alignment between how a research team member and how an adolescent would categorize a post, we also reviewed the codebook with adolescents. In a series of 5 groups, we asked 32 adolescents (15-20 years of age) who were social media users to review our codebook and conduct content analysis on 12 posts representing WAC coding categories. The results showed between 88% and 100% agreement with evaluation by youths of these posts when applying our codebook. In particular, 2 posts that focused on youth-centered codes (a cartoon representing appeal to youths and a post showing a young-appearing model) had high agreement of assessments between researchers and adolescents.

#### Data Collection

We evaluated 1 year of social media content to capture different seasons, events, and holidays using a purposeful, nonprobability approach. Our goal was to understand the breadth of WAC-related content posted by marijuana businesses. Informed by the results of our pilot coding process, if multiple posts were present on the day evaluated, we examined the last post of that day. We coded up to 1 post per day for every other day throughout 1 year.

Multimedia content of posts on Facebook and Twitter profiles were coded including text, photographs, and images (eg, memes and downloaded icons). Coding was applied to each post, and data from each post were recorded using a typed description including verbatim text quotes and written descriptions of images. The content in a single post could be coded as representative of more than 1 codebook construct. Data were recorded in a customized, password-protected FileMaker Pro 15 database (Filemaker Inc) by trained coders. To assess coder agreement, all coders assessed a 10% subsample of posts. Interrater agreement for study variables ranged from 77% (appeal to youths) to 92% (overconsumption).

#### Codebook Variables

The coding schema based on WAC regulations is described in Table 1. For each social media post, we recorded the number of Likes/Shares on Facebook and Favorites/Retweets on Twitter.

**Table 1.** Codebook Variables Representing Categories Derived From the Washington Administrative Code 314-55-155

Variable	WAC Description	Key Elements of Coding	Subcategories
Overconsumption	All messages must not contain any statement or illustration that “promotes overconsumption.”	This variable was defined to include statements about getting high (“baked”), maintaining a high for a longer time, or finding ways to increase one’s high.	NA
Curative or therapeutic benefits	All messages must not contain any statement or illustration that promotes “curative or therapeutic effects.”	Results of pilot coding: these types of statements typically represented either stress relief (which more loosely meets criteria as a curative or therapeutic effect), or treatment of conditions that clearly meet the description of curative or therapeutic benefits. This variable was divided into 2 subcategories.	Stress relief: posts with statements about marijuana use to alleviate stress or promote relaxation. Treatment of conditions: posts with statements about marijuana use as a treatment for mental health conditions such as depression, and physical health conditions such as headaches or cancer.
Appeal to youth	All messages must avoid appealing to youths, including avoiding “objects, such as toys, characters, or cartoon characters suggesting the presence of a child, or any other depiction designed in any manner to be especially appealing to children or other persons under legal age to consume marijuana; or is designed in any manner that would be especially appealing to children or other persons under 21 y of age.”	We evaluated both images appealing to youths, and images that represented youths.	Images appealing to youths: consistent with the WAC, this category included images that were animated, or that represented toys, characters, or cartoon characters suggesting the presence of a child. Images that represented youths: this category included photographs of people who looked to be younger than 21 y. For this criterion to apply, 2 coders needed to independently assess whether the image met this criterion.
Warnings	All messages must include warnings in 4 areas, including warning of the product’s intoxicating effects, to avoid driving while using marijuana, of health risks, and that the product is only for people older than 21 y.	We evaluated for the presence of each of these warnings, together as a single disclaimer and separately as individual warnings.	(1) Intoxicating effects, (2) driving while using marijuana, (3) health risks, (4) product is for persons older than 21 y, (5) all warnings together

Abbreviations: NA, not applicable; WAC, Washington Administrative Code.

Descriptive variables for the marijuana business page included the business name, geographic location, and the number of Followers. We evaluated the population of the city where each business was located using the official city website, and classified each business as being located in an urban or rural setting using US Census categories.^20^

### Statistical Analysis

Descriptive statistics were used to characterize the social media business pages of the recreational marijuana companies. For each WAC regulation category, we calculated the frequency of posts representing that category for our overall sample, as well as for each individual marijuana company. For WAC regulations regarding warnings, we calculated the frequency of posts with all 4 warnings as well as overall frequency of any warning for the overall sample, and for each individual marijuana company. To test whether engagement of social media by youths was less common on posts with warnings, Wilcoxon rank sum tests were used to compare Likes/Favorites and Shares/Retweets between posts with warnings and without warnings. All statistical comparisons were unpaired, 2-sided tests, and *P* < .05 was used to determine statistical significance.

## Results

A total of 1027 posts were evaluated from 12 marijuana business pages on Facebook and Twitter platforms representing 6 marijuana companies. The companies had between 374 and 2915 Twitter followers and between 342 and 1592 Facebook followers. Of 6 company settings, 3 (50%) were rural and 3 (50%) were urban. Table 2 contains descriptive information for these 6 marijuana companies.

**Table 2.** Recreational Marijuana Companies With Both Twitter and Facebook Followers in the State of Washington^a^

Company	Twitter Followers, No.	Facebook Followers, No.	City Setting
A	626	1275	Rural
B	490	699	Urban
C	2915	1592	Urban
D	374	564	Rural
E	864	1265	Urban
F	401	342	Rural

^a^ Followers evaluated at study inception on August 31, 2016.

### WAC Regulations

#### Overconsumption

Of the 1027 posts, 17 (1.7%) met WAC criteria for promoting overconsumption. Example posts included, “get sun baked!” and “stay high.” One post included a photograph of a cat with the caption: “We MUST smoke hard and heavy today, clouds of ganja smoke are the last and only defense we have against this CATastrophic event.” Among these 17 posts, 7 (41.2%) derived from the Twitter account of company C.

#### Curative or Therapeutic Benefits

Of the 1027 posts, 137 (13.3%) promoted curative or therapeutic benefits, including 121 (11.8%) that promoted stress relief and 16 (1.6%) that explicitly promoted treatment of medical conditions. Examples of posts promoting stress relief included, “This gives me such a carefree and relaxed attitude,” and “Mama Ida’s Indica is a chill strain that will be perfect for her day of relaxation!” Of the 121 posts that endorsed marijuana for stress relief, 105 (86.8%) were derived from company A. Examples of posts that promoted therapeutic benefits included, “#Cannabis Used To Ease PTSD” and “MJ can literally improve your pet's health.” Of the 16 posts that promoted treatment benefits, 11 (69%) were derived from company F.

#### Youth Appeal

Of 1027 posts in the appeal to youth category, 9 (0.01%) were identified as appealing directly to youths. Of these 9 posts, 8 (88.9%) met criteria as an image appealing to youths, and 1 (11.1%) was an image of a youth. An example of an image appealing to youths was a post comparing the adverse events of alcohol and marijuana on Kermit the Frog, suggesting that alcohol was more harmful and dangerous than marijuana.

#### Warnings

A warning disclaimer that addressed all 4 types of WAC warnings was present on 110 of 1027 posts (10.7%). The typical language in this disclaimer was, “This product has intoxicating effects and may be habit forming. Marijuana can impair concentration, coordination, and judgment. Do not operate a vehicle or machinery under the influence of this drug. There may be health risks associated with consumption of this product. For use by adults 21 years of age or older. Keep out of the reach of children.” Most commonly, this disclaimer was noted on the package label with a photograph of the product posted.

The use of this disclaimer addressing all the required WAC warnings was not consistent across companies or across platforms of Facebook and Twitter (Table 3). For example, 2 of the 6 companies did not address any individual WAC warnings or use the warning disclaimer on any posts. Other companies placed warnings on 1 platform but not the other. For example, Facebook posts of company E almost always (88 of 90 posts [97.7%]) used the disclaimer to address all 4 warnings. However, minimal warnings (1 of 185 posts [0.5%]) were present on Twitter posts of company E.

**Table 3.** Warning Disclaimer Use Addressing All 4 Washington Administrative Code Warnings on Social Media For Recreational Marijuana Companies

Company	Facebook Platform		Twitter Platform	
	Posts Evaluated, No.	Frequency of Warning Disclaimer Use, No. (%)	Posts Evaluated, No.	Frequency of Warning Disclaimer Use, No. (%)
A	125	0	91	0
B	18	0	28	0
C	129	9 (6.9)	119	7 (5.9)
D	90	4 (4.4)	53	0
E	90	88 (97.7)	185	1 (0.5)
F	99	1 (1.0)	0	0

Other approaches for the use of warnings were less common. In 1 post, a warning was present that stated, “you should always be careful when mixing 1 substance with another, and not just with recreational drugs, but with medicines as well.” In another post, the company described providing free transportation for people who were under the influence of marijuana. Another post included a photograph of a marijuana product with “not for kids” on the product label.

### Likes/Favorites and Shares/Retweets

The mean (SD) number of Likes/Favorites for a business post on social media of a marijuana company was 10.93 (3.3) (median [range], 2 [0-1200]). For Likes/Favorites on a post with WAC-adherent warnings, the mean (SD) number of Likes/Favorites was 3.3 (1.6) (median [range], 3, [0-11]). The results showed that the mean (SD) number of Shares/Retweets for a business post on social media of a marijuana company was 3.9 (36.9) (median [range], 0 [0-62]). The mean (SD) number of Shares/Retweets on a business post with WAC-adherent warnings was 0.19 (0.8) (median [range], 0 [0-8]). There were no statistically significant differences comparing the number of Likes/Favorites or Shares/Retweets between WAC-adherent (*P* = .31) and non–WAC-adherent (*P* = .51) posts.

## Discussion

This study evaluated the adherence to WAC regulations regarding social media posts across 6 recreational marijuana companies in the state of Washington. The results showed that these companies were active on Facebook and Twitter with many Followers, which illustrated that recreational marijuana companies can build extensive communication networks using social media. Most social media posts were consistent with WAC regulations that prohibited messages promoting overconsumption, curative or therapeutic benefits, or appeal to youths. However, for each WAC category, a different company was singled out as promoting prohibited content. Few recreational marijuana companies followed regulations for required warning messages presented on business posts, and placement of warnings by some companies was inconsistent across social media platforms.

The results showed that social media users, which may have included youths, who followed recreational marijuana business pages on Facebook or Twitter may have been exposed to at least 1 WAC category of prohibited content and may have never seen warnings about marijuana content. For example, a youth who followed the Twitter account from company C would likely have been exposed to messages promoting overconsumption. These messages may have influenced this youth’s perceived social norms of overconsumption as normal. Social norms are strongly associated with substance use intentions and behaviors.^21,22,23^ Furthermore, a youth who chose to follow more than 1 marijuana business page may have been exposed to multiple types of harmful messages about marijuana. For example, if a youth chose to follow social media accounts of companies C and F, this youth would have been exposed to content promoting overconsumption of marijuana as well as content that suggested the health benefits of marijuana use.

Industry violators who craft messages appealing to youths about a restricted substance are concerning, but perhaps not surprising in the setting of previous literature about tobacco and alcohol. Regarding messaging about alcohol use, a previous study found that violations of industry regulations were common, including targeting alcohol advertisements with strong sexual themes in magazines with a high readership of youths.^24^ Furthermore, studies have shown that the exposure of adolescents to cigarette advertisements can enhance the appeal of smoking^6,7^ and may recruit new adolescent smokers.^9,10,11^ One previous study concluded that promotions and advertisements may be a “stronger current influence in encouraging adolescents to initiate the smoking process than exposure to peer or family smokers.”^25^

Our findings described the presence of marijuana company-driven messages on social media; most youths access social media daily.^13^ The context of this study in social media is important because, whereas many forms of advertising for marijuana remain illegal, marijuana companies can use social media as an influential mode of communication with potential customers. A previous study surveyed middle school students and found that the exposure to advertisements for medical marijuana was associated with stronger intentions to use marijuana.^26^ This exposure may have ongoing influence; another study found that among young adults who had used marijuana, exposure to marijuana advertising was associated with heavier and more potent marijuana use.^27^ It is important to note that since the data collection of this study, the WAC website content describing social media guidelines has been removed. All language referencing social media within the WAC that was present at the inception of this study is no longer present. This means that the concerning content described in this study is no longer explicitly addressed by WAC regulations.

A second finding was that some marijuana companies used claims of relaxation and stress relief to promote their products, and other companies suggested curative benefits. This approach of promoting potential health benefits is in stark contrast to current tobacco and alcohol messaging, and this may lead to assumptions of marijuana as a “safe” substance. Our previous study found that the discourse surrounding legalization of marijuana led some adolescents and young adults to perceive that marijuana was safe because legalization implied it was now endorsed by the government.^28^

A third finding was that social endorsement of posts via Likes/Favorites and Shares/Retweets was common but highly variable. This result supported the potential reach of these posts. Furthermore, sharing a post on an individual’s own profile allowed the content to bypass any age restrictions, and may have increased exposure for youths who view the profiles of their peers. We found no statistically significant differences between the frequency of Likes/Favorites and Shares/Retweets on posts with warnings and those posts without warnings. This finding has important implications, as it indicated that marijuana businesses could include these warnings on social media posts via Likes/Favorites and Shares/Retweets without fears of decreasing user engagement on their social media posts.

### Limitations

Limitations to the study included our focus on companies that maintained both Facebook and Twitter business pages and had social media content present since 2015, which likely led to a biased selection of companies that had been in existence longer with greater social media experience and expertise. It is possible that our findings overrepresented compliance with WAC regulations. Our study represented a selection of posts spanning 1 year. Given our focus on representing content across a full calendar year, our approach of evaluating 1 post per day, every other day, meant that many posts were not recorded. Our approach of selecting the final post of the day led to a nonprobability sample and may have led to bias toward posts associated with happy hour or evening activities. Furthermore, we only captured publicly available content on Facebook and Twitter platforms. We did not capture other marketing strategies promoted by publications in the lay press such as private Facebook groups and direct messaging with consumers to build rapport.^8,29^ Because this content is private, it is unclear how it may differ from the findings in this study. It is also important to note that our study evaluated the frequency of posts and types of social media content promoting overconsumption, and it can be argued that in the adolescent population, there is no safe level of marijuana consumption. Future studies may consider evaluating the frequency and content of social media posts that promote any marijuana consumption to better represent content that is not appropriate for youths.

We did not anticipate a change in the WAC approach to social media, and we cannot anticipate how display patterns by marijuana companies are now different as a result of this change. However, without this approach, it is likely that prohibited content is at least as frequent on social media by marijuana companies. Alternatively, it is possible that WAC regulations were perceived as ineffective on social media, although no rationale nor data to support this change are offered.

## Conclusions

Despite these limitations, our study had important implications. Marijuana legalization remains a recent phenomenon, and policies for appropriate messaging are still developing. This ongoing development is clearly seen in the evolution of the posted WAC regulations during the course of this study. Thus, it is a critical time to shape policies to prevent exposure of youths to marijuana messaging. One possible policy approach is to enforce age gating on marijuana business pages. Age gating would limit access to the business page based on the age stated on the user’s profile. It would prevent youths who accurately represent their age on social media from following the business pages of marijuana companies.

Another possible approach is to clearly define social media messaging as advertising and apply the current WAC regulations, then enforce these regulations through monitoring and fines for violations. The WAC regulations are meant to address advertising, and it is hard to argue that a social media post encouraging the purchase and consumption of a retail product does not represent some form of advertising. However, monitoring the large amount of social media content generated daily by marijuana companies could limit the feasibility of this approach. The fines for violations could support the additional staff needed to conduct ongoing social media monitoring. For example, if a $1000 fine was paid for each incident of prohibited content identified in our study, over $160 000 would become available to the Washington State Liquor and Cannabis Board. Another potential consideration is to create a way for concerning content to be tagged by viewers and reported to social media companies, similar to approaches in social media for reporting bullying or offensive content. This approach may allow users to be part of WAC regulation enforcing, though public interest in pursuing this reporting is unknown. Future studies should evaluate these and other innovative approaches toward defining and enforcing regulations to prevent the marketing of recreational marijuana to youths.
